# Effectiveness of real-time classroom interactive competition on academic performance: a systematic review and meta-analysis

**DOI:** 10.7717/peerj-cs.1310

**Published:** 2023-04-12

**Authors:** Jose Manuel Jurado-Castro, Salvador Vargas-Molina, Jose L. Gómez-Urquiza, Javier Benítez-Porres

**Affiliations:** 1 Maimonides Biomedical Research Institute of Cordoba, University of Cordoba, Córdoba, Spain; 2 Escuela Universitaria de Osuna, Universidad de Sevilla, Osuna, Spain; 3 CIBER Fisiopatología de la Obesidad y Nutrición (CIBEROBN), Instituto de Salud Carlos III, Madrid, Spain; 4 Faculty of Medicine, University of Malaga, Málaga, Spain; 5 Faculty of Sport Sciences, EADE-University of Wales Trinity Saint David, Málaga, Spain; 6 Faculty of Health Sciences, University of Granada, Ceuta, Spain

**Keywords:** Mobile learning, Gamification, Kahoot!, Smartphone, Students, Classroom

## Abstract

In recent years, different tools have been introduced into the educational landscape to promote active participation and interaction between students and teachers through personal response systems. The evolution of this methodology has allowed students to participate in real-time by answering questions posed. Previous reviews on the effectiveness of real-time classroom interactive competition (RCIC) on academic performance have been performed; however, this research was based only on Kahoot, without considering other RCIC tools or programs. In addition, the RCIC effectiveness at different educational levels and its effect according to the duration of the intervention has not been meta-analytically analyzed until to date. The aim of this meta-analysis was to analyze the RCIC effectiveness in improving academic performance. A search focused on studies from the educational field published from 2010 until September 2022 was performed. Experimental studies with objective and valid data (scores based on tests or exams) were included. From a total of 397 studies considered potentially eligible, 23 studies met the inclusion criteria. The sample was *n* = 1,877 for the experimental group and *n* = 1,765 for the control group with an academic improvement in favor to experimental group (MD 7.34; CI [5.31–9.43]; *p* < 0.001). There was also significant improvement in academic performance when analyzing different educational levels and different tools. In addition, both short-term interventions (two weeks or less in duration) and long-term (from two weeks to one year in duration) were effective. Therefore, RCIC interventions seem to be an effective strategy to improve academic performance.

## Introduction

In recent decades, electronic devices, such as smartphones and tablets, are increasingly present in the market, as a sign of the great technological development of society, which, in addition to allowing a permanent connection to the internet, stimulate the participation and interest of students at all educational stages. One of the current challenges in teaching is optimizing class time and increasing student motivation towards the teaching-learning process. The proper use of this type of device makes them a methodological tool that could be useful for this purpose, complementing the traditional classes (Heflin, Shewmaker & Nguyen, 2017). The traditional class teaching method seems to cause a loss of concentration and passivity in the students (Miller, 2014). To overcome this problem, teachers should try to increase the level of participation of students during the development of the class, thus preventing loss of concentration in long duration sessions.

There are different strategies to making learning more interactive, such as: using audience responses, videos, audiovisual aids, written material. Moreover, strategies such as: dividing the class into groups, presenting cases where students can work, questioning the audience, organize guest talks, debates, using simulations and role plays (Snell, 1999). Nevertheless, at the beginning of the 21st century, student response systems, initially called “clickers”, burst onto the educational scene. These are systems equipped with an electronic command by means of which questions and questions are answered in real time. These systems made it possible to ask collective questions to a group-class and collect the individual responses issued by the students and show the statistical graphics of the results (Caldwell, 2007). Nowadays, these systems have evolved, being available in various formats with intuitive interfaces for any portable electronic devices, regardless of the operating system used. As a result of this evolution, and the mobile phone penetration in the population (Langford et al., 2019), many of the inconveniences that the first systems presented have been solved: the use of special equipment, the time invested in training by teachers and students, and the economic cost involved in purchasing the number of models necessary for use in a class. The game-based learning is an advancement in learning technology, it could motivate and involve players in such a way that they learn without being aware of it (Gee, 2003). Serious games can be beneficial for classroom dynamics, motivation and academic performance (Sharples, 2000). Several student response systems have introduced game features to increase student participation, such as the Space Race games in Socrative (Dervan, 2014) and Quizlet (Chien, 2015).

With the development of real-time response system applications for smartphones, there is now the ability to quickly assess student knowledge. Real-time classroom interactive competition (RCIC) is carried out with the use of platforms web-based student immediate response systems, which seems to help students learn through play, through an environment of communication and interaction that encourages feedback and reinforcement between teachers and students, and between students themselves with their peers. This initiative can incorporate a fun learning method for students (Chin, 2014). These types of platforms are generally known as mobile learning (ML). ML is widely used in the educational context, the students have access to the questions posed by the teacher through the “room or quiz number”. The administrator can generate or import multiple choice, short answer questions or true/false. These ML programs provide statistics on the answers given that can be evaluated in real time. Both the version for computer and mobile devices (smartphones and tablets) are free (although there are paid versions) and the only requirement for its use is a previous registration by the teacher. ML programs can facilitate the construction of knowledge, problem solving and the development of diverse skills and abilities in an autonomous and ubiquitous way, thanks to the mediation of portable devices (mobile phone, laptop, tablet, *etc*.) and internet (Georgiev, Georgieva & Smrikarov, 2004). Normally, the ML interface is designed with many interactive features (including music in some cases), where students *via* mobile devices can join games and answer questions, and view the answers to their choices. The applications software has been designed to involve users in activities motivated by their personal preferences (Licorish et al., 2018). This low-cost technology has been recognized as being of great value in enhancing the educational experience for teachers and students alike (Collins, 2007; Guerrero et al., 2016; Rodriguez, Ortiz & Aguilar, 2018). However, some studies consider that excessive contact with technology or use of ML could present several issues in class, such as a negative impact on teaching (Tondeur et al., 2017) and the students (Chen, Pedersen & Murphy, 2012), and there is reluctance among some teachers to use mobile devices regularly in class, claiming that they impair human communication and socialization and are potential distractors (O’Bannon & Thomas, 2015). Moreover, the use of ML could present problems related to network connectivity (Wang & Tahir, 2020). However, despite these drawbacks, this traditionalist point of view is challenged by the results of recent studies that highlight the benefit that mobile technology can bring in different aspects of the teaching-learning process (Wang & Tahir, 2020).

Previously, reviews have been carried out on gamification based on ML in the educational field (Crompton & Burke, 2018; Baptista & Oliveira, 2019; Huang et al., 2020; Noroozi, Dehghanzadeh & Talaee, 2020; Sailer & Homner, 2020; Wang & Tahir, 2020; Zhang & Yu, 2021). In relation with the use of RCIC tools or programs, a recent literature review concluded that Kahoot can positively affect learning outcomes from the perspective of interaction (Zhang & Yu, 2021). This study concluded that future research could be conducted to determine the effectiveness of Kahoot in different contexts such as primary education, higher education, and occupational training. Another review of the literature that focused solely on Kahoot concluded that it can have a positive effect on learning performance, classroom dynamics, student and teacher attitudes, and student anxiety. However, the researchers noted that there are also studies in which Kahoot has little or no effect (Wang & Tahir, 2020). Therefore, objective and specific reviews seem to be needed. In addition to extending the research to other programs or applications besides Kahoot. Many of these reviews cited above have not focused exclusively on the promotion of academic performance (getting required marks or academic scores according to the standards set) through the RCIC. Therefore, there is currently no consensus on RCIC contribution to improving academic performance, despite its recent popularity.

This article provides a systematic review and meta-analysis of the literature on the effectiveness of RCIC through mobile applications or web-based student immediate response systems platforms on academic performance. Due to the fact that there are different types or classifications of RCIC without a consensus on their effectiveness in improving academic performance, this article has the aim of shedding light and establish a consensus on the benefits of RCIC in the academic performance of students, and that these considerations can be taken into account by education professionals. Therefore, the present systematic review and meta-analysis can be of great benefit to education professionals (different academic levels) who wish to apply new methods based on the information and communication technologies and ML to improve the academic performance of their students, obtaining after reading a broad view of the effectiveness of these RCIC tools as a learning strategy. In addition, it could provide a base for researchers or developers who wish to carry out new work or create new tools or technological applications, since it seems that the applications designed by educators or researchers themselves could be effective in improving academic performance.

To ensure broad coverage, inclusion criteria based on the PICO (S) question (Moher et al., 2009) were used, performing a search in the following databases: Wiley InterScience, Science Direct, PubMed, Web of Science and ERIC. The search was based on the following terms: ‘academic performance’, ‘technological learning’, ‘classroom dynamics’ and ‘gamification’. These aspects are detailed in the manuscript.

Therefore, the present meta-analysis aimed to contribute in terms of its design to resolve the current gaps in the literature. It examined studies carried out in all academic cycles with different intervention duration (short-time and long-time). The studies were based on comparing RCIC interventions to improve academic performance with respect to control groups (CG) that did not receive a RCIC intervention, and academic performance was objectively measured. The study also aimed to contribute on which RCIC tools are the most effective for improving student performance, including popular known tools, such as Kahoot to self-designed tools by the researchers themselves, with the aim of shedding light and establish a consensus on the benefits of RCIC in the academic performance of students, and that these considerations can be taken into account by education professionals.

## Materials & Methods

A systematic review and meta-analysis was carried out ac-cording to the Preferred Reporting Items for Systematic Reviews and Meta-Analysis (PRISMA) (Moher et al., 2009; Page et al., 2021) (Supplementary Material S1). The review protocol was registered at the PROSPERO (International Prospective Register of Systematic Reviews), with the registration number CRD42020212646.

### Inclusion criteria of selected studies

The inclusion criteria of this systematic review and meta-analysis were established according to the PICO (S) outline: P (population): Students of all levels and types of education (including children and adults); I (intervention) and C (comparison): Experimental group (EG) that received intervention or evaluation with RCIC *versus* a CG; O (outcome): Improvement in academic performance; (S) (type of study): experimental studies.

Exclusion criteria were: (i) the article is not accessible in any way through university services or memberships; (ii) studies that were not reported with measurable and objective data (numerical values or scores based on a test or exam) on academic performance; (iii) articles in other languages apart from English.

### Protocol for electronic searching

The databases Wiley InterScience, Science Direct, PubMed, Web of Science and ERIC were used to execute the search query between 2010 and September 2022. These databases were considered main sources to retrieve highly quality related papers. The search query was composed in English. ML based in RCIC was queried using terms such as ‘academic performance’, ‘technological learning’, ‘classroom dynamics’ and ‘gamification’.

### Study selection and data collection

The search and analysis of the studies was conducted by two researchers (J.M.J-C. and J.B-P.). The reference manager RefWorks was used to code the articles found (Hendrix, 2004), the discrepancies regarding the interpretation of the extracted data were discussed by both investigators. Moreover, the articles were filtered manually using the inclusion criteria, an internal code for researchers was associated with each selected article.

Next, an individual analysis was conducted for each study separately (Petticrew & Roberts, 2006). The following information about each study was extracted: (a) general information (country, year of publication), (b) method (participants, educational level, measure of educational performance), (c) kind of intervention, and (d) general results and conclusions.

### Risk of bias in individual studies

According to the Cochrane Collaboration, the risk of bias was analyzed for each study (Sterne et al., 2019). Seven domains (sequence generation, allocation concealment, blinding of participants and staff, blinding of outcome assessment, incomplete outcome data, selective outcome reporting, and other aspects considered) were assessed, with a rating of high, low or unclear risk of bias.

### Statistical analysis

To perform this meta-analysis, the effect of RCIC educational interventions was examined on improvement of academic performance, comparing EG with a CG. Data were obtained using the sample size, the mean and standard deviation of the evaluation data (numerical values) presented after the intervention or a pos *t*-test. The numerical evaluation values presented in the selected studies were subjected to a multiplier to present the data over a value of 0–100 in order to standardize the results for subsequent analysis and reduce heterogeneity.

The results of the present meta-analysis were presented as forest plots with mean differences (MD) and 95% confidence interval (CI). Heterogeneity was also presented, which was calculated by measuring its scope by the *I*^2^ index. To analyze publication bias, Egger test (Egger et al., 1997) was used. A sensitivity analysis was also performed to assess that the effect size did not significantly vary after eliminating each study from the analysis. The authors examined the value of *P* for this statistic, warning of the presence of heterogeneity when *P* < 0.05, which compromises the validity of the pooled estimates (Takkouche, Cadarso-Suárez & Spiegelman, 1999). Moreover, the *I*^2^ index considered a low heterogeneity (0% to 40%); moderate (30%–60%); considerable (50%–90%); or substantial (75%–100%) (Shamseer et al., 2015). Given that heterogeneity is presumed in this study, a random-effects model method was used to measure the effect of the included studies (Berlin et al., 1989; Lipsey & Wilson, 2001; Shadish & Haddock, 1994).

Moreover, to identify the possible sources of variance in overall effect and heterogeneity, as two categorical moderators (school level and program type) were categorized in selected studies, subgroup analyses were applied. Moreover, a subgroup analyses were applied to difference the effectiveness between short-term (≤ two weeks) and long-term (≥ two weeks) interventions.

To carry out the meta-analysis, the Review Manager program was used (RevMan, computer program) version 5.4.1. was used (Cochrane, 2008). There was considered a statistical significance in all analyses with a value of *P* < 0.05. Results are shown in MD followed by CI.

## Results

### Studies selected

**Figure 1:**
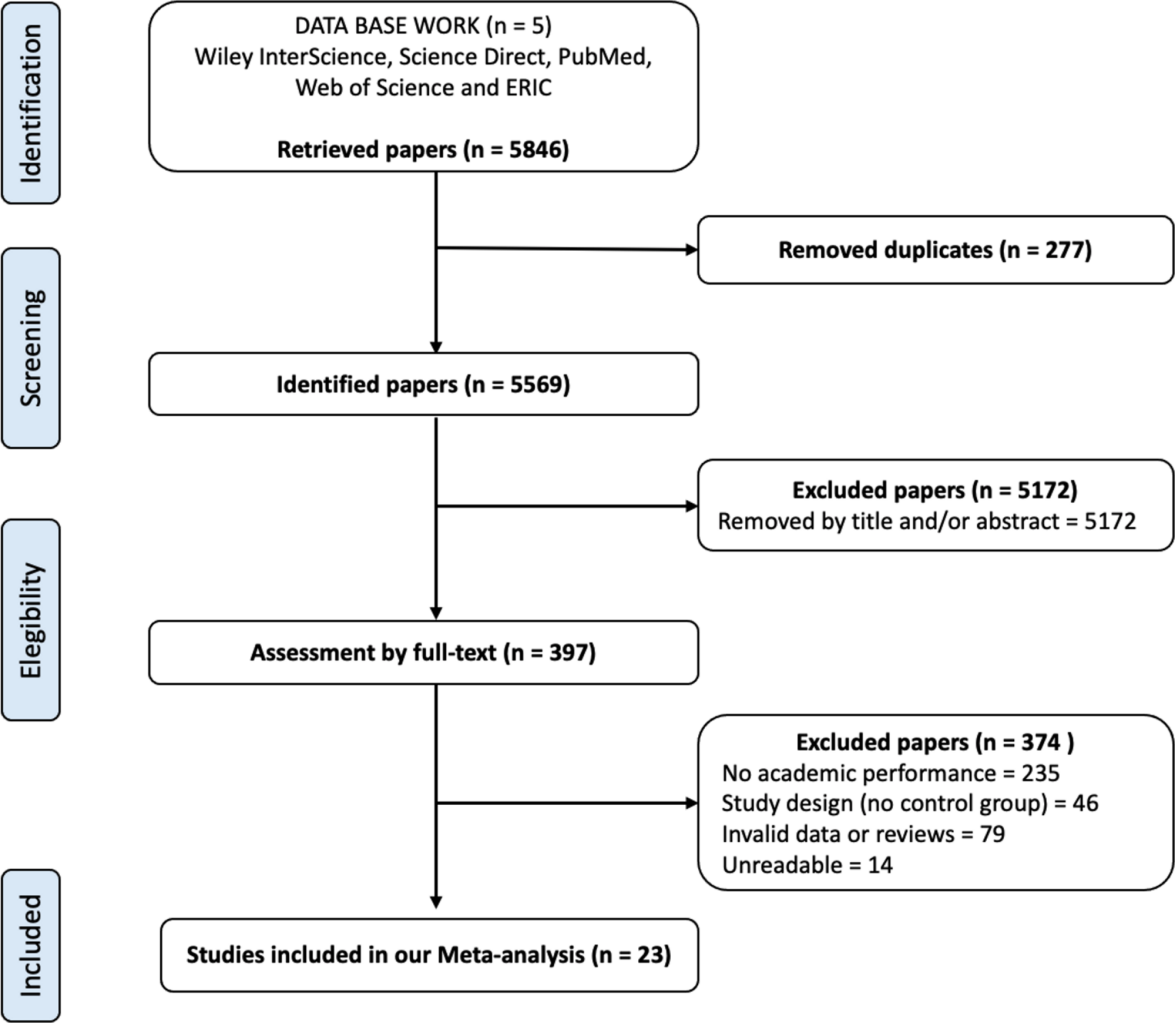
Flow diagram describing the study-identification process.

A total of 5,846 articles were identified from the included databases. 277 duplicates were eliminated, thus leaving a result after the search of 5,569 publications. According to the title and abstract, 397 publications that were potentially relevant were identified, from which the full text was extracted. After applying the inclusion and exclusion criteria, 347 studies were excluded. Therefore, 23 articles were finally selected for the present meta-analysis (Fig. 1).

### Description and characteristics of selected studies

The characteristics of the included studies are provided in Table 1. Six of selected studies were carried out in Spain, (Ferriz-Valero et al., 2020; Garcia-Cabot et al., 2019; García Iruela & Hijón Neira, 2018; Martínez-Jimenez & Ruíz-Jiménez, 2020; Pozo-Rico & Sandoval, 2019; Segura-Robles et al., 2020), five in the USA (Bawa, 2019; Kinder & Kurz, 2018; Linganna et al., 2020; Sarkar, Ford & Manzo, 2017; Shatto, L’Ecuyer & Quinn, 2017), two in Turkey (Asmali, 2018; Bilgin & Gul, 2020), two in Taiwan (Lee et al., 2019; Hung, 2016), two in Greece (Tsihouridis, Vavougios & Ioannidis, 2017; Legaki et al., 2020), and only one in the following countries: Malasya (Lai et al., 2020), Korea (Park et al., 2019), Germany (Sailer & Sailer, 2020), England (Sanchez, Langer & Kaur, 2020), Norway (Wang, Zhu & Sætre, 2016) and Thailand (Wichadee & Pattanapichet, 2018). A total of 3,642 students (22 ± 6 years) were selected in the meta-analysis, where 1,877 participated in experimental group (EG) and 1,765 in CG.

The interventions had durations ranging from less than a week to 1-academic year (Table 1). Two studies had a one academic year (Martínez-Jimenez & Ruíz-Jiménez, 2020) or a year duration (Shatto, L’Ecuyer & Quinn, 2017). The majority (*n* = 13) of selected had duration ranging 2 to 14 weeks. Five studies (García Iruela & Hijón Neira, 2018; Kinder & Kurz, 2018; Park et al., 2019; Sailer & Sailer, 2020; Wang, Zhu & Sætre, 2016) had a duration lower than a week, specifically these had a duration of 1-day (García Iruela & Hijón Neira, 2018; Kinder & Kurz, 2018; Sailer & Sailer, 2020; Wang, Zhu & Sætre, 2016) and one of them was a duration of 3-days (Park et al., 2019). Three studies not reported information relative to duration of the intervention (Legaki et al., 2020; Sarkar, Ford & Manzo, 2017; Tsihouridis, Vavougios & Ioannidis, 2017).

Seventeen of the interventions were conducted in university education (Asmali, 2018; Bawa, 2019; Bilgin & Gul, 2020; Ferriz-Valero et al., 2020; Garcia-Cabot et al., 2019; García Iruela & Hijón Neira, 2018; Hung, 2016; Kinder & Kurz, 2018; Legaki et al., 2020; Martínez-Jimenez & Ruíz-Jiménez, 2020; Park et al., 2019; Sailer & Sailer, 2020; Sanchez, Langer & Kaur, 2020; Sarkar, Ford & Manzo, 2017; Shatto, L’Ecuyer & Quinn, 2017; Wang, Zhu & Sætre, 2016; Wichadee & Pattanapichet, 2018), three in secondary school (Lee et al., 2019; Segura-Robles et al., 2020; Tsihouridis, Vavougios & Ioannidis, 2017), one in primary school (Pozo-Rico & Sandoval, 2019), and two interventions were carried out with Junior doctors (Lai et al., 2020; Linganna et al., 2020).Various subject disciplines were involved in the reviewed studies including: bussiness courses (Bawa, 2019; Legaki et al., 2020; Martínez-Jimenez & Ruíz-Jiménez, 2020; Sarkar, Ford & Manzo, 2017); English language (Hung, 2016; Park et al., 2019; Wichadee & Pattanapichet, 2018); technology or computer network systems administration (García Iruela & Hijón Neira, 2018; Tsihouridis, Vavougios & Ioannidis, 2017; Wang, Zhu & Sætre, 2016); physical education (Ferriz-Valero et al., 2020; Segura-Robles et al., 2020); medical sciences (Lai et al., 2020; Linganna et al., 2020); nursing (Kinder & Kurz, 2018; Shatto, L’Ecuyer & Quinn, 2017); tourism and hospitality (Asmali, 2018); learning environments (Bilgin & Gul, 2020); science master (Garcia-Cabot et al., 2019); educational sciences (Sailer & Sailer, 2020); psychology (Sanchez, Langer & Kaur, 2020); emotional intelligence—primary school (Pozo-Rico & Sandoval, 2019); secondary education general (Lee et al., 2019).

**Table 1:** Characteristics of selected studies by real-time classroom interactive competition intervention.

**Authors (year)**	**Country**	**Sample (*n*)**	**Age (years)**	**App or program**	**Education level**	**Career (subject)**	**Intervention duration**	**Experimental results (Score CG *vs* EG) /Total Score /*p*-value**	**Academic performance —main results**
Asmali (2018)	Turkey	43	19–22	Kahoot!	University	Tourism and Hospitality	10 weeks	46.05 *vs* 63.15/100/*p* = 0.035	Clicker use in the experimental group indicated significantly higher post-test scores for the experimental group
Bawa (2019)	USA	96	18–60	Kahoot!	University (Undergraduate)	Business Courses	7 weeks	56.83 ± 18.65 *vs* 79.56 ± 13.1/100/*p* = 0.0001	Learners’ performance and engagement are enhanced when using Kahoot versus traditional teaching methods.
Bilgin & Gul (2020)	Turkey	92	17–22	Edmodo	University	Learning Environments	13 weeks	65.1 ± 19.7 *vs* 76.4 ± 14.23/100/*p* < 0.05	No significant difference was established between the gamified and traditional groups in terms of students’ attitudes towards group learning environments and the course, the gamified group outperformed the traditional group in terms of group cohesion scores and team member evaluation scores.
Ferriz-Valero et al. (2020)	Spain	127	22 ± 3.5	ClassCraft®	University	Physical Education	5 weeks	5.44 *vs* 5.99/10/*p* = 0.001	Gamified implementation is beneficial for academic performance at the university stage
Garcia-Cabot et al. (2019)	Spain	27	25–40	Design by researchers	University	Science Master	2 weeks	8.54 ± 0.72 *vs* 9.53 ± 0.71/10/*p* = 0.0025	Gamified social e-learning platform can improve the learning performance and engagement of Science Master students.
García Iruela & Hijón Neira (2018)	Spain	24	25 ± 5.75	Kahoot!	University	Computer Network Systems Administration	2 h	6.57 ± 1.41 *vs* 8.27 ± 1.49/10/NR	Students using gamification as a means of learning improved slightly more than the rest.
Hung (2016)	Taiwan	44	20–22	Kahoot!	University	English Language Teaching	2 weeks	77.45 ± 11.35 *vs* 86.18 ± 8.61/100/*p* = 0.006	Gamified use of clickers had positive influences on student learning
Kinder & Kurz (2018)	USA	98	20–29	Kahoot!	University (Undergraduate)	Nursing	4 × 20 min sessions	88 ± 3.5 *vs* 91 ± 4/100/*p* = 0.005	Both groups showed almost same scoring, but after t test the intervention group marked higher. It was shown increasement on the knowledges after using the technology and students identified it as positive for learning.
Lai et al. (2020)	Malasya	31	27 ± 1.5	FlipQuiz	Junior doctors	Medical Sciences	8 weeks	20.27 ± 0.65 *vs* 20.28 ± 0.7/30/NR	Gamification approach could be an effective alternative to conventional approach in point-of-care ultrasonographic training.
Lee et al. (2019)	Taiwan	39	NR	Kahoot!	Secondary	General Education	6 weeks	68.8 ± 16.25 *vs* 73.26 ± 15.95/100/*p* = 0.39	Implementation of “Kahoot!” can enhance these rural-area students’ learning motivation, gain the rural-area students’ attention, and create incentives for the students to preview and review learning materials promoting learning efficiency
Legaki et al. (2020)	Greece	75	NR	Horses for Courses (Design by researchers)	University and Undergraduates students	Bussiness Administration	NR	32.3 ± 11.4 *vs* 43.2 ± 20.6/100/NR	Gamification had a positive impact on student learning compared to traditional teaching methods
Linganna et al. (2020)	USA	18	NR	EchoEducator	Junior doctors	Transesophageal Echocardiography	2 weeks	73.74 *vs* 81.82/100/*p* = 0.26	Smartphone-based asynchronous educational application improves transesophageal echocardiography knowledge compared with traditional modalities alone
Martínez-Jimenez & Ruíz-Jiménez (2020)	Spain	116	18–35	Kahoot!, Socrative or Quizziz	University	Human Resources Management and Business Administration	1 academic year	5.68 ± 1.53 *vs* 6.75 ± 1.69/10/NR	Academic results have been improved with flipping courses, compared with traditional lectures.
Park et al. (2019)	Korea	64	22.8	Design by researchers	University	English Learning	3 days	8.56 ± 4.4 *vs* 10.97 ± 3.85/25/*p* = 0.023	The results from the experiment show that the proposed reward structure produces a statistically significant increase in the level of learning,
Pozo-Rico & Sandoval (2019)	Spain	1424	8.48 ± 1.49	Game-based E-Learning	Primary	Emotional Inteligence	7 weeks	7.22 ± 1.28 *vs* 7.98 ± 0.74/10/*p* < 0.001	Implementation of Emotional Inteligence game-based e-Learning into classroom teaching effectively improved academic achievement in primary school students using both methods
Sailer & Sailer (2020)	Germany	205	22.59 ± 3.18	*Quizalize*	University	Educational Science	1 day	0.47 ± 0.2 *vs* 0.58 ± 0.21/1/NR	Gamified learning shown a positive indirect effect of gamification on application-oriented knowledge that is mediated by learning process performance
Sanchez, Langer & Kaur (2020)	England	473	NR	*Online quizzes*	University	Psychology	16 weeks	71.66 ± 13.05 *vs* 70.31 ± 13.84/100/*p* = 0.07	Higher achieving students benefited more from gamification than lower achieving students.
Sarkar, Ford & Manzo (2017)	USA	182	NR	Kahoot!	University	Bussines Courses	NR	72.7 ± 10.8 *vs* 76.1 ± 9.6/100/*p* = 0.02	Technologies can assist digital natives in the learning process and lead to better academic performance.
Segura-Robles et al. (2020)	Spain	64	15 ± 1.62	Sport gamification instruments	Secondary	Physical Education	5 weeks	51.16 *vs* 55.12/100/*p* = 0.071	Flipped learning and gamification could improve performance of Physical Education students
Shatto, L’Ecuyer & Quinn (2017)	USA	47	23–42	Kahoot!	University	Nursing Education	52 weeks	807 ± 89.5 *vs* 864 ± 109/1,000/*p* = 0.046	Results indicated that short-term retention was greater and long- term retention was significantly great in the students who were taught using flipped classroom methodology.
Tsihouridis, Vavougios & Ioannidis (2017)	Greece	67	16–17	Kahoot!	Secondary	Electrical Circuits	NR	51.72 ± 8.44 *vs* 59.93 ± 10.09/100/*p* = 0.001	According to the results, the integration of Kahoot in the teaching process improved learners’ understanding of certain concepts on electric circuits, enhanced their active participation in the lesson, motivated them towards learning and constituted a creative and fun-tool to use for teaching purposes.
Wang, Zhu & Sætre (2016)	Norway	209	NR	Kahoot!	University	Science and Technology	1 day	3.34 *vs* 3.82/7/*p* = 0.147	Not find significant learning improvement.
Wichadee & Pattanapichet (2018)	Thailand	77	18–24	Kahoot!	University	English Language	14 weeks	19.92 ± 0.39 *vs* 22.74 ± 0.39/30/NR	Students had positive attitudes towards application of digital games in language learning.

**Notes.**

CG control group EG experimental group NR not reported

The intervention of the included studies was carried out mainly with Kahoot, present in twelve studies (Asmali, 2018; Bawa, 2019; García Iruela & Hijón Neira, 2018; Hung, 2016; Kinder & Kurz, 2018; Lee et al., 2019; Martínez-Jimenez & Ruíz-Jiménez, 2020; Sarkar, Ford & Manzo, 2017; Shatto, L’Ecuyer & Quinn, 2017; Tsihouridis, Vavougios & Ioannidis, 2017; Wang, Zhu & Sætre, 2016; Wichadee & Pattanapichet, 2018). Eight studies were categorized as others (Lai et al., 2020; Bilgin & Gul, 2020; Ferriz-Valero et al., 2020; Linganna et al., 2020; Pozo-Rico & Sandoval, 2019; Sailer & Sailer, 2020; Sanchez, Langer & Kaur, 2020; Segura-Robles et al., 2020). The name of the app or program (public access programs for any user at the time the study was carried out) was specified for each study in Table 1. Three studies were carried out applying programs designed by educators or researchers themselves for private use (Garcia-Cabot et al., 2019; Legaki et al., 2020; Park et al., 2019).

Information on experimental results and academic performance evaluation between CG and GA in each of the selected studies is provided in Table 1.

### Risk of bias in included studies

Low risk of bias was obtained for the seven domains generally. A high risk of bias of random sequence generation was assessed in four studies (Kinder & Kurz, 2018; Sanchez, Langer & Kaur, 2020; Sarkar, Ford & Manzo, 2017; Shatto, L’Ecuyer & Quinn, 2017). One of these studies (Shatto, L’Ecuyer & Quinn, 2017) also presented a high risk of bias in allocation concealment. The study by (Kinder & Kurz, 2018; Kinder & Kurz, 2018), also mentioned above, presented a high risk of bias due to incomplete outcome data. The summary figure and the summary graph of the risk of bias assessment were presented as Supplementary Material (Supplementary Material S2), accompanied by specific information since seven studies were evaluated with “other” risks of bias (Bawa, 2019; Legaki et al., 2020; Martínez-Jimenez & Ruíz-Jiménez, 2020; Sailer & Sailer, 2020; Sanchez, Langer & Kaur, 2020; Segura-Robles et al., 2020; Wang, Zhu & Sætre, 2016).

### Effects of real-time classroom interactive competition interventions on academic performance

**Figure 2:**
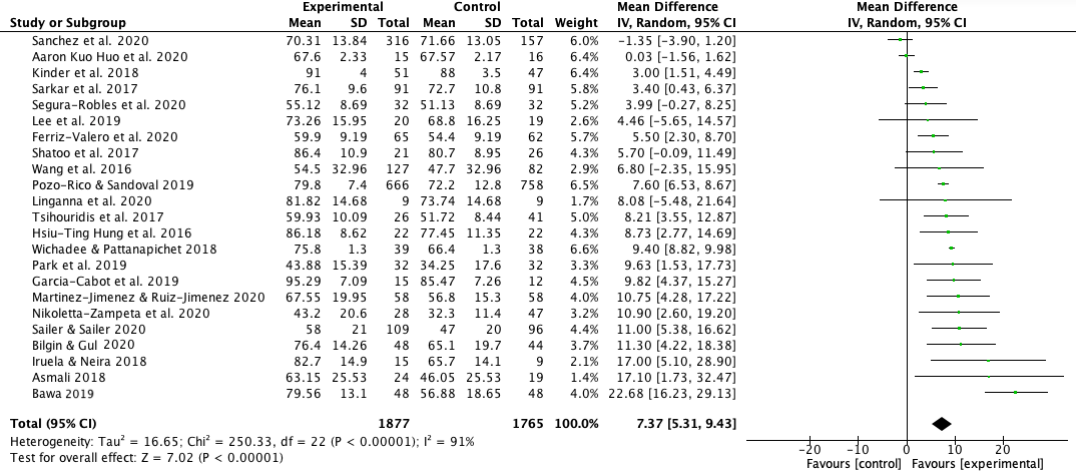
Effect interventions on academic achievement. CI, confidence interval; SD, standard deviation.

Academic performance was evaluated in the twenty-three studies selected. The total sample (students) of the meta-analysis was 3,642 (*n* = 1,877 for the EG and *n* = 1,765 for the CG). The meta-analysis showed an academic performance improvement in favor to EG (MD 7.37; CI [5.31–9.43]; *P* < 0.001; *I*^2^ = 91%) (Fig. 2). A greater learning was observed in students who used RCIC in their courses compared to those who did not.

The weight of the studies was distributed, with none of them standing out substantially above the other. The lower limit was found at 1.4% (Asmali, 2018) and the upper one at 6.6% (Wichadee & Pattanapichet, 2018), oscillating the weight of the other studies between these values. Only one study (Sanchez, Langer & Kaur, 2020) showed an effect in favor to CG (MD −1.35; CI [−3.9 to 1.2]).

To reduce the presence of heterogeneity (*I*^2^ = 91%), several moderators with random effects were used to explore possible causes. In this sense, three main categories of variables were examined: (a) school-level students; (b) type of RCIC (program or application); (c) interventions according to the time of duration. The analysis was presented in subgroups as shown below.

#### Effect of school level

**Figure 3:**
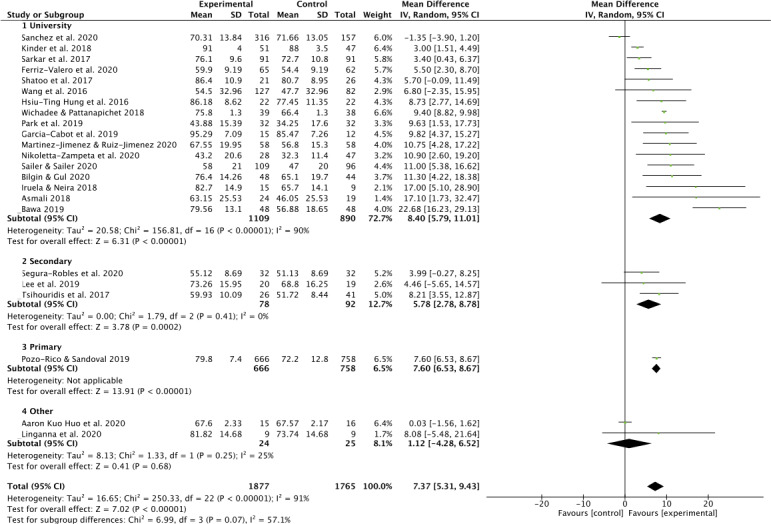
Effect interventions on academic achievement by academic level. CI, confidence interval; SD, standard deviation.

The included studied were divided in subgroups according to student’s level school: (i) University; (ii) Secondary school; (iii) Primary school; (iv) Other. The test for subgroups differences showed a valor of *I*^2^ = 57.1% displayed a moderate heterogeneity. A greater weight (72.7%) was observed for university level subgroup, followed by secondary school level (12.7%), subgroup described as “other” (8.1%), and finally primary school level (6.5%) composed only by one study (Pozo-Rico & Sandoval, 2019) (Fig. 3).

The first section included studies carried out on university students (Asmali, 2018; Bawa, 2019; Bilgin & Gul, 2020; Ferriz-Valero et al., 2020; Garcia-Cabot et al., 2019; García Iruela & Hijón Neira, 2018; Hung, 2016; Kinder & Kurz, 2018; Legaki et al., 2020; Martínez-Jimenez & Ruíz-Jiménez, 2020; Park et al., 2019; Sailer & Sailer, 2020; Sanchez, Langer & Kaur, 2020; Sarkar, Ford & Manzo, 2017; Shatto, L’Ecuyer & Quinn, 2017; Wang, Zhu & Sætre, 2016; Wichadee & Pattanapichet, 2018). A significant effect for EG was found on university level (MD 8.40; CI [5.79–11.01], *P* < 0.001; *I*^2^ = 90%). The second section included studies on secondary school level (Lee et al., 2019; Segura-Robles et al., 2020; Tsihouridis, Vavougios & Ioannidis, 2017), showed a significant effect for EG, without heterogeneity (MD 7.60; CI [6.53–8.67], *P* < 0.001; *I*^2^ = 0%). The school primary level subgroup, although there was only one study, showed an effect in favor to EG (MD 5.78; CI [2.78–8.78], *P* < 0.001) (Fig. 3). The last section, composed for the “other subgroup”, not showed effect for EG (MD 1.12; CI [−4.28 to 6.52], *P* = 0.68; *I*^2^ = 25%).

#### Effect of mobile learning program based on real-time interactive competition

**Figure 4:**
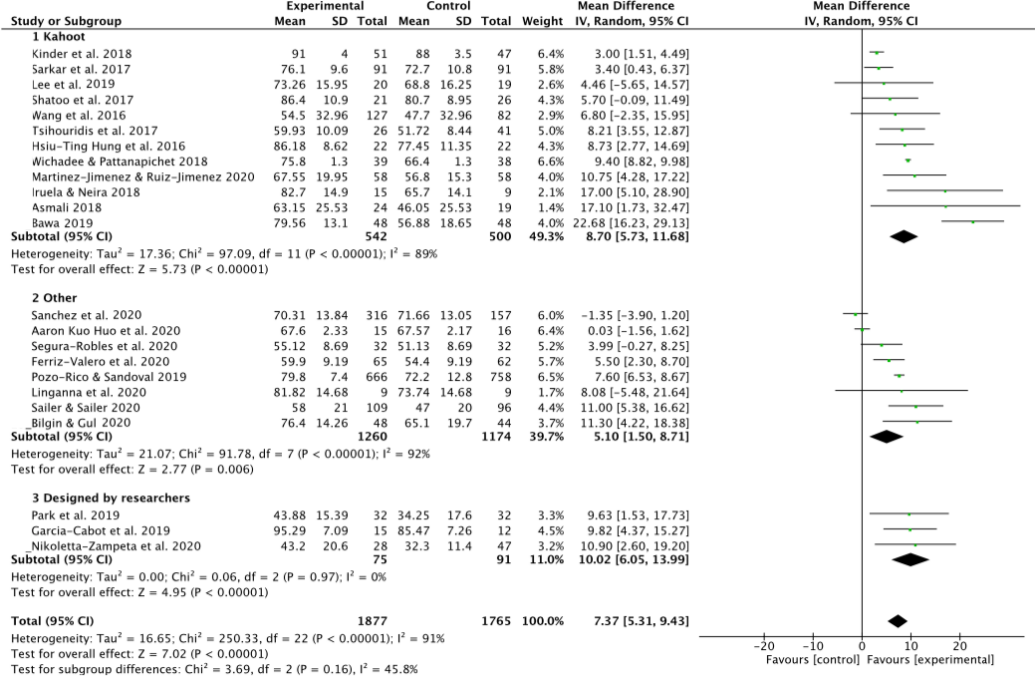
Effect interventions on academic achievement by mobile learning program based on real-time interactive competition. CI, confidence interval; SD, standard deviation.

Another subgroup classification was realized, in this case the studied were divided in subgroups according to program used: (i) Kahoot; (ii) Other; (iii) Designed by educators or researchers themselves. The test for subgroups differences showed a valor of *I*^2^ = 45.8% displayed a moderate heterogeneity. A greater weight (49.3%) was observed for Kahoot subgroup, followed by other programs (subgroup described as “other”) (39.7%), and finally ML designed by researchers (11%). In this subgroup classification, heterogeneity was only substantially reduced in the group “designed by researchers”, where was observed a *I*^2^ index = 0%. (Fig. 4).

The first section included studies realized with Kahoot (Asmali, 2018; García Iruela & Hijón Neira, 2018; Hung, 2016; Kinder & Kurz, 2018; Lee et al., 2019; Martínez-Jimenez & Ruíz-Jiménez, 2020; Sarkar, Ford & Manzo, 2017; Shatto, L’Ecuyer & Quinn, 2017; Tsihouridis, Vavougios & Ioannidis, 2017; Wang, Zhu & Sætre, 2016; Wichadee & Pattanapichet, 2018). A significant effect for EG was found for Kahoot (MD 8.70; CI [5.73–11.68]; *P* < 0.001; *I*^2^ = 89%). The second section included studies with other programs (Bilgin & Gul, 2020; Ferriz-Valero et al., 2020; Lai et al., 2020; Linganna et al., 2020; Pozo-Rico & Sandoval, 2019; Sailer & Sailer, 2020; Sanchez, Langer & Kaur, 2020; Segura-Robles et al., 2020), showed a significant effect for EG (MD 5.10; CI [1.5–8.71], *P* < 0.001; *I*^2^ = 92%). The greatest overall effect was obtained with the programs designed by educators or researchers themselves (Garcia-Cabot et al., 2019; Legaki et al., 2020; Park et al., 2019), showed a significant effect for EG, without heterogeneity (MD 10.02; CI [6.05–13.99]; *P* < 0.001; *I*^2^ = 0%) (Fig. 4).

#### Real-time interactive competition effectiveness according to the intervention time

**Figure 5:**
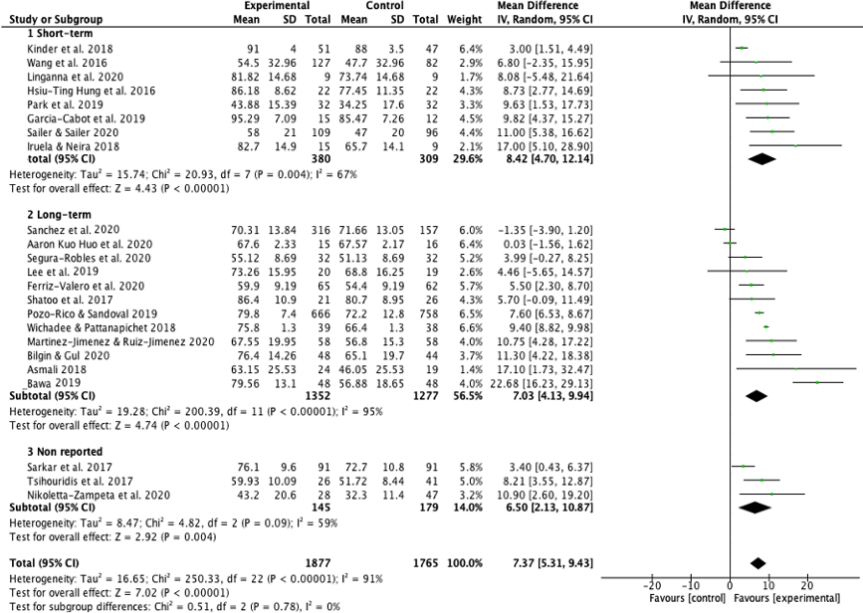
Effect interventions on academic achievement by short-term and long-term real-time interactive competition interventions. CI, confidence interval; SD, standard deviation.

Eight studies (Garcia-Cabot et al., 2019; García Iruela & Hijón Neira, 2018; Hung, 2016; Kinder & Kurz, 2018; Linganna et al., 2020; Park et al., 2019; Sailer & Sailer, 2020; Wang, Zhu & Sætre, 2016) were classified as short-term interventions, while twelve studies (Asmali, 2018; Bawa, 2019; Bilgin & Gul, 2020; Ferriz-Valero et al., 2020; Lai et al., 2020; Lee et al., 2019; Martínez-Jimenez & Ruíz-Jiménez, 2020; Pozo-Rico & Sandoval, 2019; Sanchez, Langer & Kaur, 2020; Segura-Robles et al., 2020; Shatto, L’Ecuyer & Quinn, 2017; Wichadee & Pattanapichet, 2018) were classified as long-term interventions. Three studies (Legaki et al., 2020; Sarkar, Ford & Manzo, 2017; Tsihouridis, Vavougios & Ioannidis, 2017) did not indicate the duration of the intervention (Fig. 5).

Short-term interventions obtained a significant effect in favor to EG (MD 8.42; CI [4.70–12.14]; *P* < 0.001; *I*^2^ = 67%), in the same way it occurred with long-term interventions (MD 7.03; CI [4.13–9.94]; *P* < 0.001; *I*^2^ = 95%).

## Discussion

The present meta-analysis based on quantitative studies measured by objective test scores comparing CG *vs* EG, provided evidence that academic RCIC interventions carried out were effective to improve student academic performance. Based on 23 RCIC interventions and a total of 3,642 students, our study found that students in the EG scored an average of 7.37% higher on exams than those in the CG. The EG obtained a higher score (8.4%) when the interventions were carried out at the university, compared to the rest of academic cycles, a higher score (10.02%) when the interventions were carried out with ML tools designed by the researchers or educators themselves, and with better results (8.42%) when these were carried in a short term of less than weeks.

Despite the popularity of ML based on RCIC, there is no consensus on whether it contributes to improving academic performance or not. According to a recent meta-analysis in the pre-publication phase based on RCIC interventions with Kahoot (Yu, 2021), has been shown to significantly improve the academic performance of students. The present meta-analysis examined whether the effect of RCIC with different tools, beyond that Kahoot, improved the academic performance in the students.

This meta-analysis found that the RCIC interventions did not differ across grade levels on academic performance. This finding shows that this type of intervention could be a design applicable from elementary school to university level. In this context, three studies are at the secondary education level and only one study is at primary education, so more studies are required to clearly establish the effect of gamification to improve academic performance in primary and secondary schools. Despite the different subgroups, the results of our meta-analysis of subgroups by academic levels showed a greater effect in improving academic performance for gamification studies carried out with university students, in addition to being the most numerous in our selection of studies, and if there is a wide literature on the matter, in different areas or subjects (Andreu, 2020; Domínguez & Bezanilla, 2019). In addition to academic performance, positive results have been found in the motivation of university students with the implementation of gamification experiences in the classroom (Andreu, 2020).

Based on the inclusion criteria of our study, other interventions were found that did not belong to these academic levels, however they were based on education and training for the performance of their employment. This subgroup classified as “others” was made up of the Lai et al. (2020) study, which relied on gamification in ultrasound training for beginning physicians, and by Linganna et al. (2020), also carried out with young doctors based on an asynchronous educational application based on smartphones for the knowledge of transesophageal echocardiography compared with traditional modalities alone. These “others” interventions did not have a significant effect.

Different programs or tools were described to carry out RCIC interventions, although the most common one so far in the literature, as well as in the studies included in our work, has been Kahoot. According to the results of our meta-analysis, the RCIC tools self-designed by the researchers themselves were more effective with a greater difference in means in the score of the EG group on the CG, followed by Kahoot, and the studies that included other programs in their interventions, despite this all tools had a significant improvement in improving academic performance. The success of self-designed tools compared to the others is surely due to the fact that the interface and the gamification environment were completely adapted to the specific needs of the students. Among the most widely accepted mechanics in the reviewed literature, the scoring systems, fictitious or real gifts or rewards, ranking, achievements, avatars, stamps (badges), unlocks of new skills, levels, missions or challenges stand out team or alone.

Study interventions selected a duration ranging from less than two weeks to 1 academic year. Both short-term and long-term interventions showed a significant effect. The fact that short-term interventions tend to have a greater MD score (EG *vs* CG) compared to long-term, 8.42 *vs* 7.03, respectively, raises the problem of the ‘novelty effect’.

An important aspect to consider is that the novelty effect seems to be a conditioning factor in the success of short-term interventions, because the participants are subjected to something new to them (Clark, 1983). In this regard, gamification used in the short-term could lead to high participation by students, and may provide a greater academic achievement, therefore, the novelty effect could be a confusing variable on the student academic achievement in gamification interventions (Jeno et al., 2019). In relation to long-term intervention, an aspect to take into account related to the Cowan study (Cowan, 2010) that argued that long-term interventions can lead to superficial understanding and learning, because the ability to memorizing of each student is highly variable. Lai et al. (2020) study, selected in our meta-analysis, carried out an evaluation after the intervention and at two months post-intervention, without finding differences in the evaluation after the test with those of the 2 months after the training, which suggests that there was good retention of knowledge and skills.

Students, especially university students, regularly use their smartphones in the educational center on many occasions as a communication and entertainment tool. This can be detrimental to student learning in class. However, students feel comfortable using smartphones as a pedagogical tool.

Another important aspect to consider is that the integration of ML elements does not guarantee its effectiveness. Efficacy depends on different factors such as how to implement gamification (Hamari, Koivisto & Sarsa, 2014), as well as learner characteristics and student’s context (Buckley & Doyle, 2017). It requires significant effort and good planning in order to match gaming elements with instructional objectives. The objectives of gamification must be clear, making sure that the proposals respond to the needs raised. Students must understand the scoring system. The profile and interests of the student body, the class size, and the teaching and learning approaches that are being implemented to accommodate different types of students from different disciplines should be taken into account (Andreu, 2020).

According to the results of this meta-analysis, the use of RCIC can help increase academic performance and the effectiveness of learning in the classroom. Based on previous research, the use of certain RCIC applications, such as Kahoot it is very effective and helps the learning process in the classroom The Kahoot app can be used effectively for gamification lessons. The application of gamification with this medium can have an impact on students that makes them more ambitious and motivated to learn (Lin, Ganapathy & Kaur, 2018). The use of the RCIC in the learning process can enrich the quality of student learning in the classroom, with the greatest influences reporting on class dynamics, participation, motivation and the improvement of learning experiences (Bicen & Kocakoyun, 2018). Another study (Andreu, 2020) has suggested that the learning styles and the dynamics and mechanics used during gamification may influence differently depending on the type of students. One of the keys to integrating gamification is the context of learning and play on which it is based. So, gamification appears to have a positive effect on learning compared to traditional approaches.

The present study has some limitations. First, the level of heterogeneity is high, which has been controlled with different analysis of categorical possible moderators. In addition, in terms of subgroup analysis, there were subgroups with a reduced number of articles per category, this was due to the limited literature that met the inclusion criteria, for example, in interventions carried out in the primary cycle education or tools of self-designed ML based on RCIC, so the results in the present meta-analysis should be interpreted with caution.

Second, the studies come from different countries, with their own education system characteristics and this factor should be taken into account. Future research should evaluate, if the combination of different ML based on RCIC tools can increase the improvement in academic performance. In this sense, there is a gap in the literature

## Conclusions

This meta-analysis supports the notion that RCIC interventions seem to be successful in academic performance. There was a significant improvement in academic performance in the different educational cycles, as well as with the use of different tools. In addition, both short-term interventions and long-term were found to be effective.

Education professionals and teachers can consider the results obtained in this meta-analysis as practical application. Firstly, they should know that interventions based on RCIC seem to be effective in improving students’ academic performance. Secondly, they should consider that the interventions have proven to be effective in all educational cycles, especially in university students, with the exception of instruction on young workers related to specific topics of medical sciences. Third, the tools self-designed by the educators seem to be effective for improving academic performance. Self-designed ML based on RCIC allows adapting to the specific educational needs of students, both in student motivation and educational learning. Finally, both short-term (less than weeks) and long-term (up to one academic year) interventions seem to be effective for improving academic performance, although considering that, in short-term interventions, the students have obtained greater success on the final score.

Finally, we believe that this study contributes to an objective meta-analytic understanding of the RCIC effectiveness to improve student academic performance. The consolidation of communities is beneficial to student retention and academic engagement. Consistent technological advancements have facilitated the design of innovative tools and approaches with potential applications in education. For instance, virtual reality devices, augmented reality, and massively multiplayer online systems represent such tools. The integration of these technologies with relevant learning theories incorporating an RCIC system within the classroom environment can promote students’ educational outcomes and academic performance across diverse educational levels, surpassing current methods, as the tools used by the studies included in our meta-analysis. Consequently, it is recommended that future research investigate the feasibility of these new technological tools within an RCIC based system to establish their efficacy in enhancing academic performance. Additionally, future research could expand to examine more components such as cognitive loads, satisfaction, or anxiety with the use of RCIC.

##  Supplemental Information

10.7717/peerj-cs.1310/supp-1 Supplemental Information 1 PRISMA checklist

10.7717/peerj-cs.1310/supp-2 Supplemental Information 2 Risk of bias

## Additional Information and Declarations

### Data Availability

The following information was supplied regarding data availability: The raw measurements are available in Table 1.
