# Supplementary material for: Effectiveness of real-time classroom interactive competition on academic performance: a systematic review and meta-analysis
Source: PeerJ Comput Sci. 2023 Apr 12;9:e1310. doi: 10.7717/peerj-cs.1310 (PMC10280400; doi:10.7717/peerj-cs.1310)
Supplement: Supplemental Information 2 [file peerj-cs-09-1310-s002.docx]

**Supplementary material 2**


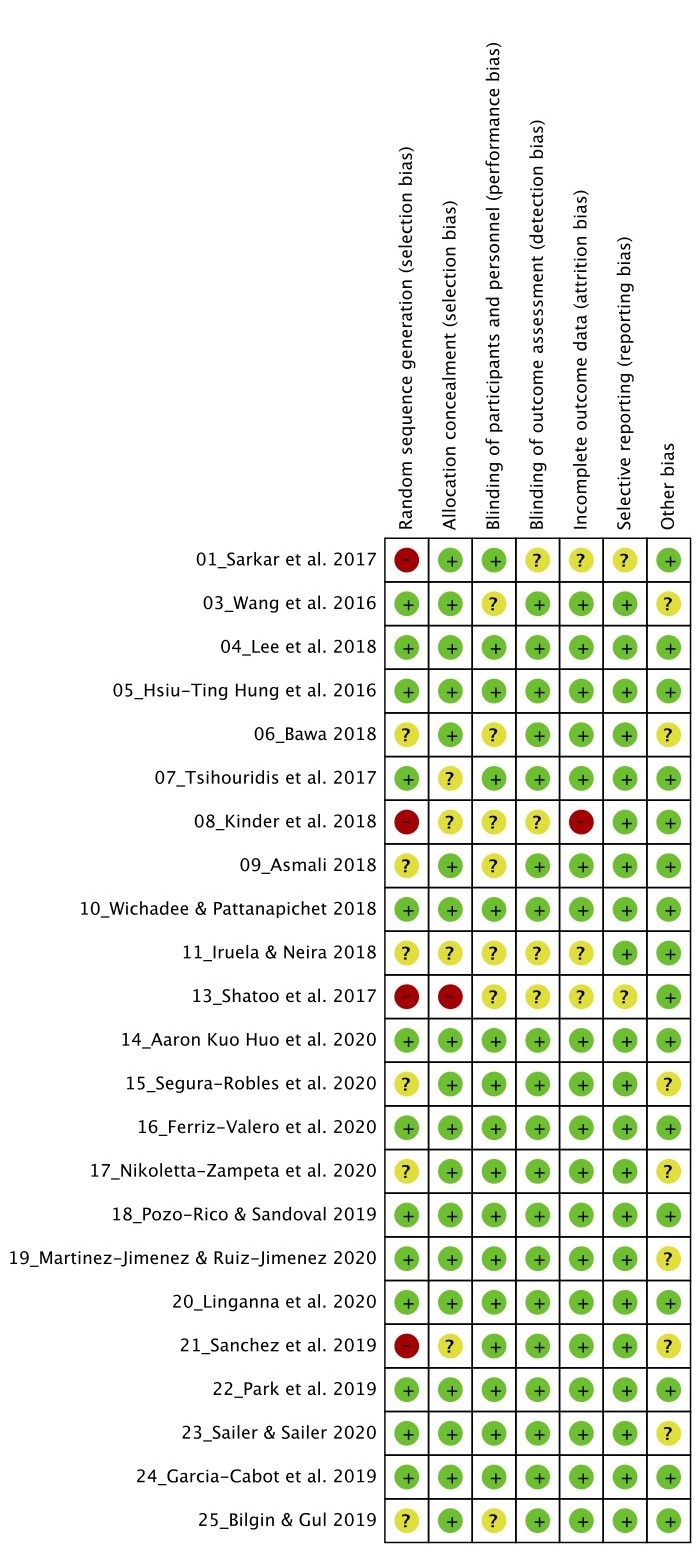


**Other bias description:**

3_Wang et al. 2016
Difference in the sample between very large groups, one group 125 participants, another 175 and that of kahoot 82

6_Bawa 2018
That instructors want to use Kahoot can convey a more positive result to students than those who did not use it.

15_Segura-Robles et al. 2020
The selection of the sample was carried out through an intentional sampling due to the ease of access to the students

17_ Nikoletta-Zampeta et al. 2020

The treatments did not have the same duration as the tasks.

19_ Martinez-Jimenez & Ruiz-Jimenez 2020

The questionnaire was voluntary and anonymous.

21_Sanchez et al. 2019
There was a very unbalanced sample, 157 gamified and 317 traditional and there was no randomization in the tests

23_Sailer & Sailer 2020

The participants were asked to prepare the videoconference, and from there an initial evaluation was made, it is not possible to control what each subject has studied at home.
